# Gallbladder agenesis: A case report and review of the literature

**DOI:** 10.1016/j.ijscr.2018.10.061

**Published:** 2018-11-01

**Authors:** Irakli Pipia, George Kenchadze, Zaza Demetrashvili, Grigol Nemsadze, Lika Jamburia, Tamari Zamtaradze, Ivane Abiatari

**Affiliations:** a Institute of Medical Research, Ilia State University, 3/5, Cholokashvili Ave., 0162, Tbilisi, Georgia; b Department of Surgery, Kipshidze Central University Hospital, 29, Vazha-Pshavela Ave., 0160, Tbilisi, Georgia; c Department of Surgery, Tbilisi State Medical University, 33, Vazha-Pshavela Ave., 0177, Tbilisi, Georgia; d Department of Radiology, Tbilisi State Medical University, 33, Vazha-Pshavela Ave., 0177, Tbilisi, Georgia; e Department of Radiology, Kipshidze Central University Hospital, 29, Vazha-Pshavela Ave., 0160, Tbilisi, Georgia

**Keywords:** Gallbladder agenesis, Laparoscopy, MRCP

## Abstract

**Introduction:**

Gallbladder agenesis is a rare congenital abnormality with an incidence of 10–65 per 100,000. Approximately half of these patients are surgically operated on because of the symptoms similar to biliary colic, and correct diagnosis is established intra-operatively.

We present a clinical case of gallbladder agenesis from our practice.

**Presentation of case:**

A 49 (forty-nine) - year- old women was admitted in the Emergency Department of our clinic. Symptoms were similar to the biliary colic. Ultrasonography showed hyperechogenic acoustic shadow on the projection of the gallbladder which was considered as constricted gallbladder and cholecystolithiasis was diagnosed**.** Laparoscopic cholecystectomy was considered. During laparoscopy gallbladder could not be found. Surgical operation was completed without conversion. Postoperative treatment included analgesics and antispasmodics. Pre-operative symptoms disappeared. One month later magnetic resonance cholangiopancreatography (MRCP) confirmed gallbladder agenesis diagnosis. Health condition of the patient is satisfactory, without any complications after a year of surgery.

**Discussion:**

Gallbladder agenesis presented with symptoms similar to biliary colic can be diagnosed without surgical intervention. Conservative treatment consists of antispasmodic drugs.

**Conclusion:**

If the shrunken gallbladder is detected on the ultrasound, additional radiological examinations are required. MRCP is considered as a test of choice among the radiological investigations. If gallbladder agenesis is identified on laparoscopy, there is no need for further conversion. For postoperative follow up examination MRCP investigation is recommended.

## Highlights

• Gallbladder agenesis presented with symptoms similar to biliary colic can be diagnosed without the need for surgical intervention.
• MRCP is considered a test of choice for diagnosis of gallbladder agenesis.
• If gallbladder agenesis is discovered during laparoscopy no conversion to laparotomy is needed.

## 1 Introduction

The work is reported in line with the Surgical Case Report Guidelines (SCARE) criteria [1]. Gallbladder agenesis is a rare congenital abnormality with an incidence of 10–65 per 100,000. Females are more commonly affected than males: frequency ratio – 3:1. Half of the patients suffer from symptoms which are similar to biliary colic. This is the reason why they are frequently operated mistakenly and confirmation of correct diagnosis occurs only during operation [[2], [3], [4], [5]]. All above-described underlines the importance of high-quality and precise testing in order to avoid unnecessary surgery.

We present a clinical case of gallbladder agenesis from our practice.

## 2 Presentation of case

A 49 (forty-nine)- year- old women was admitted to the Emergency Department of our clinic. Symptoms were similar to the biliary colic: pain in the right upper quadrant of abdomen radiating to the right shoulder and nausea. Fever and jaundice were not present. From the anamnesis we learned that similar complaints were present twice: 6 months before and 3 weeks before current presentation and that time the symptoms had been alleviated after conservative treatment. Patient was haemodinamically stable.

**Fig. 1:**
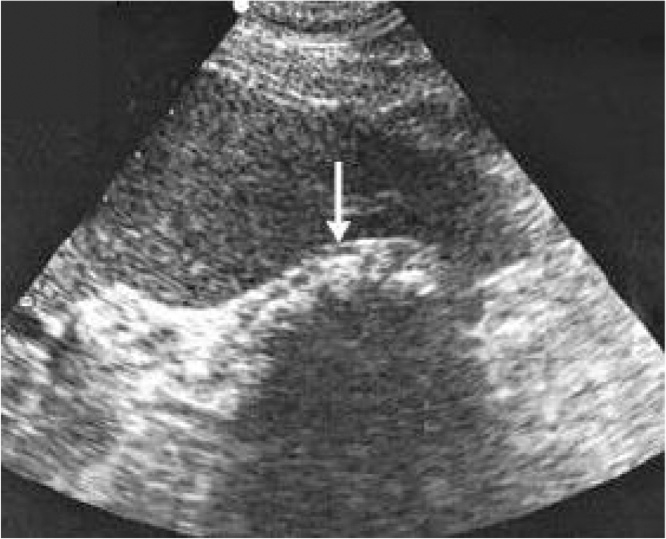
Ultrasound shows a constricted gallbladder with the possible acoustic shadow in its projection.

**Fig. 2:**
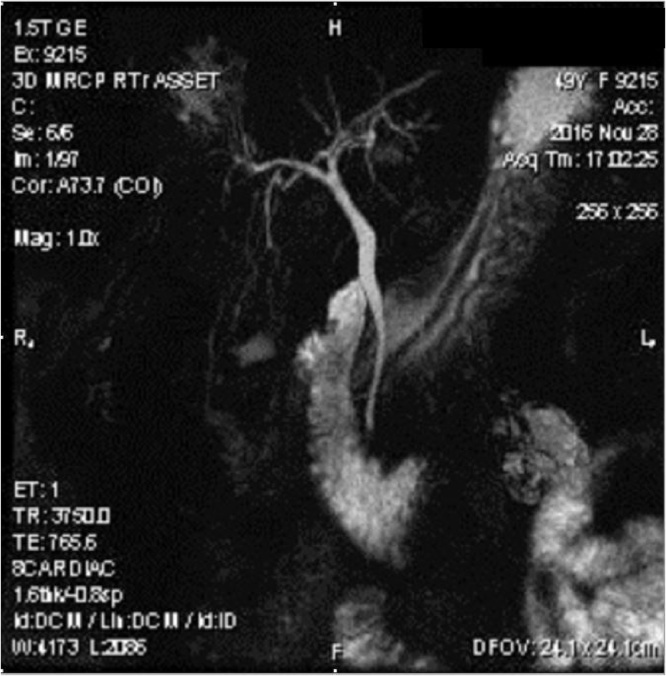
MRCP image shows normal intrahepatic and extrahepatic bile ducts, absence of gallbladder and cystic duct.

Objective clinical examination revealed tenderness in the right superior quadrant of the abdomen, slight tension of the muscles and positive murphy’s sign. Blood test results were within the reference range. Ultrasonography showed hyperechogenic acoustic shadow on the projection of the gallbladder which was considered as constricted gallbladder (Fig. 1). No pathologies were observed in the biliary ducts and cholecystolithiasis was diagnosed. Laparoscopic cholesystectomy was considered. During laparoscopy gallbladder could not be found. We performed complete visualization of the hepatic and common bile duct from the confluence of right and left hepatic ducts until common bile duct disappeared behind the second part of the duodenum. Gallbladder, cystic artery and cystic duct could not be found. The surgical operation was completed without conversion. No postoperative complications were present. Administered postoperative treatment included analgesics and antispasmodics. Pre-operative symptoms disappeared. One month later magnetic resonance cholangiopancreatography (MRCP) confirmed gallbladder agenesis diagnosis (Fig. 2). Health condition of the patient is satisfactory, without any complications after a year from the surgical operation.

## 3 Discussion

First case of Gallbladder agenesis was described in 1701 by Lemery [3,6]. Since then, there have been described about 500 similar clinical cases in the medical literature. Most of them are presented with single clinical cases [3,5]. The disease is rare and congenital, with an incidence of 10–65 per 100,000 [2,4]. But the research papers, which are based on the evaluation of autopsy material, shows increased incidence till 90 per 100,000 [7]. Bennion et al. described three groups of presentation of gallbladder agenesis: the first group presents the symptoms similar to biliary colic (pain in the right upper quadrant of the abdomen, dyspepsia), 50% of cases of gallbladder agenesis are assigned in this group. The second group consists of asymptomatic anatomical abnormalities seen incidentally on autopsy. 35% of patients are united in this group. And the third group (15% of whole number) presents gallbladder agenesis associated with the severe fetal anomalies [6].

During Gallbladder agenesis the mechanism of biliary colic is unknown, but greater part of authors consider that it is determined by dysfunction of sphincter oddi and biliary dyskinesia. The fact that pain is relieved after antispasmodic treatment strengthens this theory [2,4,8]. In our case the patient also underwent antispasmodic therapy also and during one year after surgery she continues to be asymptomatic.

In 50% of the patients with gallbladder agenesis, who complained of the similar symptoms to biliary colic, correct diagnosis: gallbladder agenesis is diagnosed intraoperatively [3,5,[8], [9], [10], [11]]. Preoperative diagnosis is often complicated. The main reason of diagnostic difficulties is a “constricted, shrunken gallbladder” and sometimes hyperechogenic shadows seen by radiologist under ultrasonography. Those shadows are considered as gallbladder stones and patient is mistakenly diagnosed with cholelithiasis and later is operated too [3,9,[11], [12], [13], [14]]. Our case repeats this typical mistake. Is it possible to avoid unnecessary surgery and can gallbladder agenesis be diagnosed before surgery? Yes, of course, it is possible with help of the modern diagnostic techniques. There exists a diagnostic and management algorithm of gallbladder agenesis published by Malde in 2010 for the cases which is manifested with the similar symptoms as biliary colic [12]. According to this algorithm, it is necessary to perform another radiological investigations: MRCP, computed tomography (CT), Endoscopic retrograde cholangiopancreatography (ERCP), Endoscopic ultrasound if it is impossible to visualize gallbladder under the ultrasound or if it is shrunk. The diagnosis of gallbladder agenesis is helped by above listed examinations, which could be treated conservatively. From all of these methods, we prefer MRCP, taking into consideration its’ informative character in the diagnosis of gallbladder agenesis [2,[15], [16], [17]]. We think that other three methods should be chosen if MRCP could not be performed.

There exists one more debatable question: should the laparoscopic operation be converted into the open surgery if no gallbladder could be found during laparoscopy? According to the opinion of quite big amount of surgeons no conversion is needed, because laparoscopy allows performing a complete and high-quality visualization of the abdominal cavity [[3], [4], [5],^9^,^11^,^14^]. They consider that conversion to the laparotomy lengthens the duration of surgery, makes it more traumatic for the patient and increases the risk of postoperative complications. In their opinion, postoperative radiological investigations are needed in order to confirm pre-existing diagnosis. The other group of surgeons considers the need of switching from laparoscopy to the laparotomy during surgery [13,18]. We belong to the first group of researchers. In our opinion, no conversion from laparoscopy to laparotomy is needed and MRCP in the postoperative period is optimal decision for such patients. We followed to this opinion in case of our patient.

## 4 Conclusion

Gallbladder agenesis presented with symptoms similar to biliary colic can be diagnosed without the need for surgical intervention. If the shrunken gallbladder is detected on the ultrasound, additional radiological examinations are needed. MRCP is considered as a test of choice among existing radiological methods. Conservative treatment consists of antispasmodic drugs. If gallbladder agenesis is discovered during laparoscopy, no conversion to laparotomy is needed. MRCP is the first choice diagnostic method in such cases during postoperative period also.

## Conflicts of interest

The authors declare that they have no conflict of interests.

## Sources of funding

This research did not receive any specific grant from funding agencies in the public, commercial, or not-for-profit sectors.

## Ethical approval

The ethical approval has been exempted as it was not necessary in the case report by our institute.

## Consent

Written informed consent was obtained from the patient for publication of this case report and accompanying images. A copy of the written consent is available for review by the Editor-in-Chief of this journal on request.

## Author contributions

Zaza Demetrashvili participated in the design of case report, developed the literature search and wrote the manuscript. Irakli Pipia, George Kenchadze, Tamari Zamtaradze and Ivane Abiatari developed the literature search and assisted in writing up. Grigol Nemsadze and Lika Jamburia carried out the radiological examination and assisted in writing up. All authors read and approved the final manuscript.

## Registration of research studies

N/A. It is a case report.

## Guarantor

Zaza Demetrashvili.

## Provenance and peer review

Not commissioned, externally peer reviewed.
